# When snacks become meals: How hunger and environmental cues bias food intake

**DOI:** 10.1186/1479-5868-7-63

**Published:** 2010-08-25

**Authors:** Mitsuru Shimizu, Collin R Payne, Brian Wansink

**Affiliations:** 1 Department of Applied Economics and Management, Cornell University, 110 Warren Hall, Ithaca, NY, 14850, USA; 2 Department of Marketing, New Mexico State University, 310 Guthrie Hall, Las Cruces, NM, 88012, USA

## Abstract

**Background:**

While environmental and situational cues influence food intake, it is not always clear how they do so. We examine whether participants consume more when an eating occasion is associated with meal cues than with snack cues. We expect their perception of the type of eating occasion to mediate the amount of food they eat. In addition, we expect the effect of those cues on food intake to be strongest among those who are hungry.

**Methods:**

One-hundred and twenty-two undergraduates (75 men, 47 women; mean BMI = 22.8, *SD *= 3.38) were randomly assigned to two experimental conditions in which they were offered foods such as quesadillas and chicken wings in an environment that was associated with either meal cues (ceramic plates, glasses, silverware, and cloth napkins at a table), or snack cues (paper plates and napkins, plastic cups, and no utensils). After participants finished eating, they were asked to complete a questionnaire that assessed their hunger, satiety, perception of the foods, and included demographic and anthropometric questions. In addition, participants' total food intake was recorded.

**Results:**

Participants who were in the presence of meal-related cues ate 27.9% more calories than those surrounded with snack cues (416 versus 532 calories). The amount participants ate was partially mediated by whether they perceived the eating occasion to be a meal or a snack. In addition, the effect of the environmental cues on intake was most pronounced among participants who were hungry.

**Conclusions:**

The present study demonstrated that environmental and situational cues associated with an eating occasion could influence overall food intake. People were more likely to eat foods when they were associated with meal cues. Importantly, the present study reveals that the effect of these cues is uniquely intertwined with cognition and motivation. First, people were more likely to eat ambiguous foods when they perceived them as a meal rather than a snack. Second, the effect of the environmental cues on intake was only observed among those who were hungry.

## Background

There is considerable evidence that environmental and situational cues influence food intake [1]. However, the specific psychological (cognitive and motivational) processes underlying those relationships are not often well addressed. This research illustrates how cognitive and motivational factors mediate and moderate the relationship between environmental cues and food intake. Specifically, consider whether a person views an eating occasion - such as a reception or a party - as a snack or meal. Typically, a meal involves a particular set of characteristics (such as eating while seated, with utensils, and so on) [2,3], and they generally involve greater calorie intake than snacks [2,4]. If people perceive the occasion as a meal, their hunger may lead them to increase their consumption - via goal fulfillment - more than if they had instead perceived it to be a snack. If environmental cues that suggest an eating occasion is either a meal or a snack can lead to an increase or decrease in consumption, then people's perception of such should mediate the relationship between these cues and how much they eat.

Yet, there is sparse research examining the possibility of environmental cues affecting food perceptions in this context. One exception is a study conducted by Pliner and Zec [5]. In Experiment 1 they found that participants who ate foods in appetizer-main course-dessert order in a meal-like environment (seated at a dining table in a carpeted room) were more likely to describe the experimental condition using meal or lunch type words than those who ate the same foods (e.g., soup, Turkey sandwich) divided into 29 small portions in a snack-like environment (standing at a kitchen counter). For instance, participants in the snack-like condition consumed 6 portions of soup, whereas those in the meal-like condition consumed a single portion. Although the presentation of foods differed between the two conditions, the quantity of foods consumed was almost identical (i.e., 369 kcal). In Experiment 2 they demonstrated that participants who had eaten in the meal-like environments consumed less food after a 20-minute delay than those in the snack-like condition. This suggests that simply perceiving an eating occasion to be a meal made a person less likely to eat a short time later. However, because they did not assess the difference in the perceptions in Experiment 2, it is unclear if perceptions truly drove the subsequent decrease in consumption among participants in the meal-like environment. These studies also varied both the environmental cues (being seated versus standing) and the presentation of the same foods. It would be useful to keep the presentation of the food constant and to assess psychological processes underlying behavior.

What is needed is research that provides identical but ambiguous foods to people, but manipulates the surrounding environmental cues to suggest that it is either a snack or a meal. As such, changes in consumption could be more clearly attributed to the external cues from the environment, not to the food. We expect to see participants in the meal condition not only perceive those identical foods to be a meal, but also to eat more as a result. In addition, not only do we expect that this perception will mediate their intake, but we believe that the effect of the environmental cues (i.e., increased food intake) may be moderated by their level of hunger. Consistent with previous findings that people were more likely to drink when they were subliminally primed with drinking-related words only if they were thirsty [6,7], we expected that the meal-cue participants would eat more than the snack-cue participants, particularly if it had been a long time since they had previously eaten, using this as a proxy for hunger.

In summary, as research assessing psychological processes underlying the relationship between environmental cues and intake is sparse, we examine both a cognitive mediator--perception of identical foods cued as either a snack or meal--and a motivational moderator--hunger. We randomly assign undergraduate students to two experimental conditions in which they are instructed to eat ambiguous foods in an environment that is associated with either meal cues (ceramic plates, glasses, silverware, and cloth napkins at a table), or snack cues (paper plates and napkins, plastic cups, and no utensils). We expect meal-cue participants to consume more than snack-cue participants. We also expect the association between the environmental cues and the amount of food eaten to be mediated by the extent to which the eating occasion is perceived as a meal or a snack. Finally, we expect the strength of the association to be moderated by hunger, such that the association is particularly strong among those who are hungry.

## Methods

### Participants

One-hundred-twenty-two undergraduate students (75 men, 47 women), with a mean BMI of 22.8 (*SD *= 3.38), were recruited at a large northeastern U.S. university through sign-up sheets in seven large classes in fields outside of psychology and nutrition. In exchange for participation, students received extra credit and their name was entered into a drawing to win an iPod. The study had Institutional Review Board approval, and participants were treated in accordance with American Psychological Association guidelines.

### Procedure and Materials

To determine what foods would be ambiguous enough to be considered either meal or snack foods, a focus group of 120 participants rated 36 foods regarding how they perceived them as snack versus meal foods on a 9-point scale (1 = snack; 9 = meal). We used three foods that were uniform in size, could be discretely counted, and that fell in the middle of this range: quesadillas (4.04), pizza (5.35), chicken wings (4.81). To examine what cues individuals associate with a specific type of eating occasion, a pilot survey asked the same pool of participants the extent to which they associated 20 different factors (ceramic plates, seating arrangements, paper cups, plastic silverware, cloth napkins, and so on) with meals and snacks[8].

To examine whether the presence of these meal-related cues was stronger than the influence of time, four study sessions were conducted at a time traditionally associated with meals (12:00) and one at a time typically associated with snacks (3:30). After assessing their availability, participants were allocated to either a 12:00 or 3:30 session. Participants were randomly assigned to either a meal-cue condition or to a snack-cue condition at both times. An average of 30 participants were scheduled for each session. Participants were given a nametag and told it was to promote socialization.

In the meal-cue condition, participants walked into the room where the tables were already set with place settings that included a ceramic plate, a drinking glass, and silverware wrapped in a cloth napkin. After participants were seated and had an opportunity to socialize, they were told that they could then serve themselves from the buffet at their leisure, and that they could take as much food as they would like. In addition to water and diet soft drinks, three target foods were served (quesadillas, pizza, and chicken wings). While participants selected their food, researchers unobtrusively recorded how many pieces of each food were taken. After participants finished their food, and after a sufficient amount of socializing had occurred (consistent with the cover story), they were given a questionnaire to complete. Following this, they were thanked, debriefed, and dismissed. After leaving, any of the three foods remaining were separately weighed. Participant's total caloric intake was calculated by taking the difference in weight between what they served themselves and what remained.

The procedure in the snack-cue condition was identical except that the setting was altered to promote snack-like environmental cues. The dinnerware (plates and napkins) were paper, the utensils and glasses were plastic, and there was no place for participants to sit until after they finished eating.

In the questionnaire, participants were asked to estimate the total calories they believed they ate. They were then asked to indicate how much of each food they took on a 9-point scale, (1 = not very much; 9 = a lot). Satiety was measured by a two-item 9-point scale ("I couldn't eat another bite of food" and "at this moment I feel full"), which were combined into a single index of satiety given high reliability (*α *= .73). They then indicated how meal-like or snack-like they felt the foods they ate during the experiments were (1 = more of a snack; 9 = more of a meal). Last, they were asked how long since they had eaten their last meal along with demographic and anthropometric questions (gender, age, weight, and height).

## Results and Discussion

Because there were no significant main effects or interactions with gender, age, and BMI, analyses were collapsed across those variables. There was a marginally significant main effect of the time of experiment (noon versus 3 p.m.); participants who participated in the noon sessions ate directionally more than those in the 3 p.m. sessions *F *(1, 118) = 2.97, *p *= .09. However, because this main effect was only observed on their actual food intake, and because the interaction effects between this and the experimental manipulation did not reach conventional level of significance (*p *= .15), analyses were collapsed across the time of experiments. This factor was subsequently treated as a covariate in the analyses.

### The Impact of Meal-Related Cues on Intake

As expected, an analysis of covariance (ANCOVA) in which the independent variable was the experimental condition (a dichotomous variable; meal-cue versus snack-cue) and the covariate was the time of experiment, demonstrated that participants in the meal-cue condition were more likely to report that the food they ate was a meal (*M *= 3.91, *SD *= 2.08) than those in the snack-cue condition (*M *= 3.13, *SD *= 1.73), *F *(1, 110) = 4.45, *p *= .04, *η^2 ^*= .04. As indicated in Table 1, the meal-cue participants' actual caloric intake was significantly greater (*M *= 531.79, *SD *= 246.92) than the snack-cue participants' (*M *= 416.39, *SD *= 192.92), *F *(1, 119) = 7.62, *p *= .007, *η^2 ^*= .06, and they also reported eating more (all *p*s < .05). Despite eating more and reporting they ate more, there were no differences in their perceived level of satiety (*p *= .98).

**Table 1:** How meal versus snack cues influence food intake, estimated food intake, and satiety

	Snack-cues		Meal-cues		*F*-Value
Variable	*M*	*SD*	*M*	*SD*	
Actual total food intake in calories	416.39	192.92	531.79	246.92	7.62**
Estimated total food intake in calories	488.53	323.26	700.18	444.93	8.36**
					
"How many quesadillas did you take?"^1^	3.13	1.81	4.04	2.06	6.04*
"How many pieces of pizza did you take?"^1^	2.46	1.70	3.54	2.17	7.43**
"How many chicken wings did you take?"^1^	4.16	2.07	4.93	2.20	4.11*
					
Composite Satiety Index	3.95	2.40	3.92	1.99	.001

*p < .05. **p < .01 All *p*-values are 2-tailed

^1 ^Not very much = 1; A lot = 9

### How Meal versus Snack Perceptions Mediate Intake

To examine if these differences in food intake were mediated by participant's perceptions of an eating occasion being a meal or a snack, a series of multiple regression analyses were conducted to determine if the strength of the association was reduced after controlling for the participants' food perception [9]. First, we predicted each dependent variable from (a) the experimental condition (a dichotomous variable; meal-cue versus snack-cue) and (b) the time of the experiment (a dichotomous variable; noon versus 3:30 p.m.) as a controlling factor. Second, we predicted participants' perception of the foods they ate from (a) the experimental condition and (b) the time of the experiment, as in the first step. Third, we predicted each dependent variable from (a) the experimental condition (b) the time of the experiment, and (c) their perception of the foods, to see if the experimental condition was still a significant predictor for the dependent variable.

**Figure 1:**
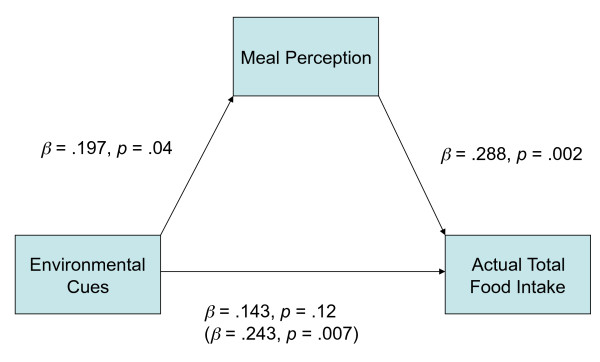
**Mediational role of the meal perception between the environmental cues and actual total food intake**.

These analyses revealed that the participants' meal perception partially mediated the association between the environmental cues and the *actual *total food intake, whereas it did not mediate the associations with other variables (such as estimated total food intake). Specifically - as needed for evidence of mediation - the significant main effect of the experimental condition in the first step, *β *= .243, *t *(119) = 2.76, *p *= .007, was not significant in the third step, *β *= .143, *t *(109) = 1.58, *p *= .12 (see Figure 1). However, because a Sobel test indicated that the mediational role of the meal perception was marginally significant, *Z *= 1.756, *p *= .08 [10], we are reluctant to conclude that the perception accounted fully for the original association between the condition and food intake. Nevertheless, in addition to conceptually replicating Pliner and Zec's findings [5], the present study showed tentative evidence of the mediational role of the perception between environmental cues and food intake.

### The Moderating Influence of Hunger

**Figure 2:**
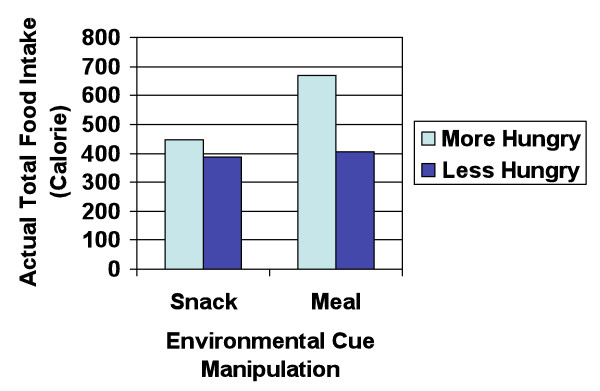
**Actual total food intake as a function of the experimental condition and motivation**.

Last, to determine if the association between the experimental condition and participants' actual food intake was moderated by their hunger, operationally defined as the length of time since they had eaten their most recent meal, we conducted a multiple regression in which we predicted participants' actual food intake from (a) the experimental condition, (b) their hunger (i.e., the length of time since they had eaten last meal), (c) the time of the experiment as a controlling factor, and (d) a condition × hunger interaction term. In this analysis, we mean-centered the scores that went into these terms by subtracting the appropriate mean from each predictor (i.e., each main effect) before computing the interaction terms [11]. This analysis revealed that there was a significant interaction effect between the experimental condition and their hunger, *β *= .217, *t *(117) = 2.30, *p *= .02. As indicated in Figure 2, simple slopes tests demonstrated that participants who were hungry (+1 *SD *above the mean) consumed much more food when surrounded with meal cues, *β *= .487, *t *(117) = 3.73, *p *< .001, than did those who were not hungry (-1 *SD *below the mean), *p *= .72. Thus, part of the impact that these effect of environmental and situational cues have on a person's food intake depends on their hunger.

Thus, consistent with general findings of the relationship between cognition and deprivation-reducing behavior [6,7], the significant impact of hunger confirms the role of motivation as a moderator. That is, the influence of environmental cues on eating behavior was observed only among participants who were hungry. This was further confirmed by an additional finding that the association between the experimental condition and participant's satiety was also moderated by hunger, *β *= .005, *t *(117) = 2.89, *p *= .005. Simple slopes tests revealed that hungry participants were more satisfied when they were in the meal-cue condition, *β *= 1.296, *t *(117) = 2.17, *p *= .03, than in the snack-cue condition. This could be because hungry participants were satisfied because they could eat more. Indeed, controlling for their actual intake reduced the association, *β *= 1.048, *t *(116) = 1.66, *p *= .10, suggesting that intake mediated the association between the experimental condition and their satiety. In contrast, those who were not hungry were actually less satisfied when they were in the meal-cue condition, *β *= -1.244, *t *(117) = -2.18, *p *= .03, which is virtually unchanged after controlling for their actual intake, *β *= -1.267, *t *(116) = -2.22, *p *= .03.

## Conclusions

Evidence is accumulating to suggest that specific meal and snack patterns influence overall food consumption, nutrient intake, and diet quality [2,12-14]. For example, Kerver et al. found that people who ate three meals per day (breakfast, lunch, and dinner) had higher intakes of micro-nutrients such as calcium, vitamins, and folic acid than those who skipped breakfast or lunch. On the other hand, breakfast or lunch skippers who ate more than two snacks had higher intakes of energy on average than those who ate three meals, suggesting that eating snacks contributes to higher consumption of energy and lower quality diets [13]. It is important to note, however, that whether a person perceives an eating occasion as a meal or a pre-dinner snack could influence what and how much they eat, and whether they decide to eat later [5]. This may be especially true for ambiguous foods such as finger foods (sandwiches, pizza, and so on) that can be perceived as either meal or snack foods.

The present study demonstrated that environmental and situational cues associated with an eating occasion could influence overall food intake. Regardless of the time of the day, people were more likely to eat ambiguous foods when they were associated with meal cues such as being seated at a table with a ceramic plate, a glass, and silverware wrapped in a napkin. Importantly, the present study reveals that the effect of these cues is intertwined with cognition and motivation. First, people were more likely to eat more of these foods by perceiving them as a meal rather than a snack. Second, the effect of the environmental cues on intake was only observed among those who were hungry. Thus, the present study not only addressed how perception of type of eating occasion mediates the association between environmental cues and food intake, but revealed that the impact of this association depended on hunger.

The first finding is particularly important in that it helps fill a research gap between the effects of environmental cues and eating behavior. Although there is substantial research evidence indicating that food intake is influenced by environmental and situational cues such as portion size [1], the role cognition plays in this relationship has not been well addressed. Although there are several potential psychological mediators between environmental cues and our food intake, the present study revealed that our perception of whether an eating occasion is a meal could play a role in the relationship.

This is not to say that people are normally aware that they eat more foods because they perceive them as meal [15]. Indeed, most evidence suggests that people are usually not aware of environmental or situational cues that influence their food intake [16,17]. It seems that because our actual food intake is unconsciously influenced by the environmental cues surrounding an eating occasion, it is only partially mediated by our conscious perception of the eating occasion.

On the other hand, given the marginally significant Sobel test, there could be other mediators between the experimental condition and actual food intake. For example, participants in the meal-cue condition may have felt more comfortable while eating, which could also influence food intake. Future research should focus on those other mediators to more thoroughly examine the relationship between the environmental cues and food intake. More importantly, the experimental design of the present study does not exclude the potential reverse causal effect. Namely, participants may have been more likely to perceive the eating occasion as meal because they ate more. Indeed, the association between the experimental condition and participants' perception of the foods was not significant after controlling for their actual total food intake, *β *= .138, *t *(109) = 1.51, *p *= .13. However, a Sobel test indicated that the mediational role of the actual food intake was not significant, *Z *= 1.619, *p *= .11, suggesting that this reverse causality also lacked the evidence of full mediational role. Future research should also examine if the meal versus snack perception influences *subsequent *food intake. For instance, it may by useful to measure whether participants perceive an eating occasion as snack or meal before they start eating. If the meal perception still mediates food intake, reverse causality can be ruled out.

The general nature of these findings has practical implications. For instance, because people who are more likely to perceive an eating occasion as a snack tend to eat less, their overall calorie intake should be smaller than those who are more likely to perceive an eating occasion as a meal. This is consistent with evidence that eating several small meals is better than eating a few big meal to decrease calorie intake [18]. However, recall that breakfast or lunch skippers who ate more than two snacks had higher intakes of energy, but lower quality diets with respect to nutrient intake [13]. The present study may suggest that those types of people tend to eat less when they perceive an eating occasion with ambiguous foods as snacks rather than meals. However, they could have subsequently eaten a full meal afterwards because they believed the snack was not enough or did not "count" as a real meal, as suggested by other studies [5]. For instance, if an individual perceives the sandwich and brownie they eat at a 5:30 reception as a snack, he or she may be more likely to follow this up with a pizza for a 7:30 dinner. This pattern can lead to substantial energy intake. Based on our findings, one possible way to prevent this is to associate those ambiguous foods with meal cues. For instance, if one eats sitting down during the reception, they may perceive it to be a meal, thus pre-empting their belief that they need to eat the 7:30 pizza.

The second finding - the moderating influence of hunger - is also important because it shows that the effect of environmental and situational cues on food intake is particularly pronounced among hungry people. In other words, people are less likely to be influenced by environmental cues in an eating setting if they are not hungry. This is conceptually consistent with previous findings suggesting the moderating role of motivation in the relation between the priming and drinking behavior [6,7]. After all, regardless of whether a person perceives a sandwich and a brownie as a meal or a snack, they need to have the physiological drive - the appetite - to consume it. We interpret this result with caution, because we relied on participants self-report of time since last meal as a proxy measure of hunger. However, the fact that hungry participants consumed a similar amount of food as those who were not hungry when they were in the snack-cue condition has a particularly important implication for reducing and preventing overeating. Given the fact that subtle environmental and situational cues influence how much people eat, changing those cues may lead to reduction in overall food intake [1,19]. As suggested in the present study, asking people to eat foods while standing may reduce consumption by cutting a snack-like environment. This reduction in consumption may reduce overeating as long as people do not compensate at a later time.

## Competing interests

The authors declare that they have no competing interests.

## Authors' contributions

MS has contributed to the analysis, interpretation, and drafting the manuscript. CRP has contributed to the design, collection, and revising the manuscript. BW has contributed to the design, collection, and interpretation of data and revision of the manuscript. All authors read and approved the final manuscript.
